# Optimization of SHIP1 Inhibitors for the treatment of Alzheimer’s disease

**DOI:** 10.1002/alz.087553

**Published:** 2025-01-09

**Authors:** Cynthia D Jesudason, Peter Bor‐Chian Lin, Disha Soni, Bridget M Perkins, Audrey Lee‐Gosselin, Cynthia M Ingraham, Will Hamilton, Emily R Mason, Omar El Jordi, Sarah Souza, Marlene Jacobson, Jerry Di Salvo, Brent Clayton, Shaoyou Chu, Jeffrey L. Dage, Adrian L Oblak, Timothy I. Richardson

**Affiliations:** ^1^ LGenia, Fortville, IN USA; ^2^ Indiana University School of Medicine, Indianapolis, IN USA; ^3^ Evotec, Princeton, NJ USA

## Abstract

**Background:**

SHIP1 is a phosphatidyl inositol phosphatase encoded by INPP5D, which has been identified as a risk gene for Alzheimer’s disease (AD). SHIP1 is expressed in microglia, the resident macrophage in brain. It is a complex, multidomain protein that acts as a negative regulator downstream from TREM2. SHIP1 possesses a phosphatase (Ptase) domain flanked by a pleckstrin‐homology (PH) domain that binds phosphatidylinositol (3,4,5)‐trisphosphate[PI(3,4,5)P_3_] and a C2 domain that binds phosphatidylinositol (3,4)‐bisphosphate [PI(3,4)P_2_]. The Ptase domain converts PI(3,4,5)P_3_ to PI(3,4)P_2_. SHIP1 also has an SH2 domain that binds to ITIMs and ITAMs where it competes with kinases. Inhibiting SHIP1 is hypothesized to have potential therapeutic benefits, as it may improve TREM2‐mediated microglial responses to neurotoxins and promote an overall neuroprotective microglial phenotype to maintain a more resilient brain and slow the rate of cognitive decline in AD patients.

**Method:**

The IUSM Purdue TREAT‐AD Center recently evaluated SHIP1 inhibitors and proposed 3‐((2,4‐Dichlorobenzyl)oxy)‐5‐(1‐(piperidin‐4‐yl)‐1H‐pyrazol‐4‐yl)pyridine for target validation studies. Structurally related analogs were synthesized and tested for SHIP1 enzyme inhibition, AKT signaling, and microglia activation in a high‐content imaging assay using HMC3 and BV2 microglia‐like cell lines. Primary microglia were treated with an optimized SHIP1 inhibitor, and subsequent changes in fibril Aβ uptake and cell viability were assessed. The NanoString nCounter Neuroinflammation assay was used to measure transcriptomic profiles. For comparison primary microglial derived from both wild‐type and Inpp5d‐haploinsufficient mice were assessed.

**Result:**

Novel SHIP1 inhibitors have been discovered and preliminary Structure Activity Relationship (SAR) studies have been completed. These compounds have shown positive results for biochemical activity, target engagement and cellular pharmacology. Both Inpp5d deficiency and pharmacological inhibition increase amyloid uptake and cell viability in primary microglia. Elevated ERK and AKT phosphorylation, after amyloid exposure, were decreased by Inpp5d deficiency. Functional pathways associated with phagocytosis, apoptosis, cytokine production, and complement system activity were altered.

**Conclusion:**

These data demonstrate that SHIP1 inhibition promotes amyloid uptake through the complement system. SHIP1 inhibition also enhances cell survival and homeostasis in primary microglia. Further studies of SHIP1 inhibition and INPP5D knockdown in animal models may provide a potential therapeutic strategy for Alzheimer’s disease.

